# Seniors’ self-preservation by maintaining established self and defying deterioration – A grounded theory

**DOI:** 10.3402/qhw.v11.30265

**Published:** 2016-05-10

**Authors:** Jeanette Källstrand Eriksson, Cathrine Hildingh, Nina Buer, Hans Thulesius

**Affiliations:** 1 School of Health and Welfare, Halmstad University, Sweden, Halmstad, Sweden; 2 School of Medical Sciences, Örebro University, Örebro, Sweden; 3 FoU Kronoberg, Växjö, Sweden

**Keywords:** Elderly, fall risk, seniors, grounded theory, living independently, self-preservation, visual impairment

## Abstract

The purpose of this classic grounded theory study was to understand how seniors who are living independently resolve issues influenced by visual impairment and high fall risk. We interviewed and observed 13 seniors with visual impairment in their homes. We also interviewed six visual instructors with experience from many hundreds of relevant incidents from the same group of seniors. We found that the seniors are resolving their main concern of “remaining themselves as who they used to be” by *self-preservation*. Within this category, the strategies *maintaining the established self* and *defying deterioration* emerged as the most prominent in our data. The theme *maintaining the established self* is mostly guided by change inertia and includes *living the past* (retaining past activities, reminiscing, and keeping the home intact) and *facading* (hiding impairment, leading to avoidance of becoming a burden and to risk juggling). *Defying deterioration* is a proactive scheme and involves *moving* (by exercising, adapting activities, using walking aids, driving), *adapting* (by finding new ways), and *networking* by sustaining old support networks or finding new networks. *Self-preservation* is generic human behavior and modifying this theory to other fields may therefore be worthwhile. In addition, health care providers may have use for the theory in fall preventive planning.

The elderly population has been growing for many years and is estimated to increase even more worldwide because of an increasing life expectancy. Therefore, organizations such as the World Health Organization (WHO) and the European Union have been articulating strategies focusing on promoting health globally among the elderly (Organization for Economic Co-operation and Development, 2006; Swedish National Institute of Public Health, 2007). Aging may be a time of self-development and self-realization where health promotion and knowledge about healthy aging are needed. It is known that healthy lifestyle behaviors, leisure activities, and social networks enhance life expectancy (Rizzuto, Orsini, Qiu, Wang, & Fratiglioni, 2012).

Being old is associated with health risks and even seniors themselves think that advanced age is equivalent to risk (Alftberg, 2012; Lundin, 2007). Even though seniors are highly heterogeneous, they are often regarded as a homogeneous group in the Western world today (Swedish Institute of Public Health, 2007). One of the major health determinants identified among the elderly is injuries, and the second most prevalent cause of injury-related hospitalizations is falls (Doran et al., 2013; Gyllensvärd, 2009; Hartholt et al., 2010). Falls and fall-related injuries are frequent among those living independently, in hospital settings, and in nursing and residential care (Gyllensvärd, 2009; McLure, Turner, Peel, & Spinks, 2005; Organization for Economic Co-operation and Development, 2007). Even if as many as 30–60% of community-dwelling elderly fall each year (Gyllensvärd, 2009; McLure et al., 2005; WHO, 2007), a fall does not always lead to injuries that require health care, but it may be a threat to an individual's quality of life (Gyllensvärd, 2009; Roe et al., 2008). Deterioration in quality of life caused by accidental falls is estimated to cost almost twice as much as direct costs such as healthcare (Gyllensvärd, 2009). Many elderly people consider falls an unavoidable and unpredictable result of the aging process and accept the constantly present risk of falling (Roe et al., 2008; Yardley, Donovan-Hall, Francis, & Todd, 2006). In an Australian interview study of 333 elderly living in the community, the majority perceived themselves as having a low risk of falls (Hill et al., 2011).

According to the National Institute for Clinical Excellence (NICE, 2004), visual impairment is one of the strongest predictive risk factors of falling, both as an independent factor and in combination with other factors. Increasing age is an independent predictor of visual impairment and ocular diseases (Gunnlaugsdottir, Arnarsson, & Jonasson, 2008). A natural decline in visual ability occurs around the age of 65 years and is perceived as impaired accommodation and/or a decline in ability to adapt when illumination suddenly changes, often caused by opacities in the lens (Laitinen et al., 2005; Rosenbloom & Morgan, 1993; Whiteside, Wallhagen, & Pettengill, 2006).

For efficient fall prevention, it is important to get the individual's perspective, since “top-down” approaches limit and restrict the lives of individuals (Yardley et al., 2006). A major issue in fall prevention is psychological attitude barriers. The elderly may either perceive fall prevention advice as authoritarian and patronizing, or as common sense and therefore of no importance (Yardley et al., 2006). Others consider themselves as non-fallers even if they actually have fallen several times. Other barriers are unfamiliarity with fall prevention if they have not experienced a fall or if they see a fall as a natural part of aging, as well as a social stigma targeting seniors as a vulnerable group (McInnes & Askie, 2004; Roe et al., 2008). Therefore, it is vital to get a deeper understanding of senior citizens’ personal main concerns when resolving daily life activities influenced by reduced visual ability and risk of falling.

## Purpose

The purpose of this study was to generate a grounded theory to explain how seniors living independently in the community resolve issues influenced by visual impairment and risk of falling. The research question according to grounded theory was, *what is going on in the lives of the studied group of people?*

## Methods

We used classic grounded theory (CGT) methodology according to Glaser in this study (Glaser, 1978, 1998). The aim of CGT is to generate conceptual theories answering the question *what is going on?* in a particular field of study. The goal is to unravel an ongoing core behavioral pattern of individuals trying to resolve a main concern. This pattern is eventually identified, named, and explained in a rich conceptual way (Glaser, 1998). The CGT researcher should trust the data and the emergence of the main concern (Glaser, 2011). The behavioral patterns are named and used in the development and write-up of a theoretical model emerging from the data by constantly and systematically comparing the data without any preconceptions (Glaser, 1998).

The theory generation has four basic steps: sampling data, coding data, writing memos, and sorting memos (Glaser, 1978, 1998). There is constant movement back and forth between the steps, and the characteristics of the steps may change depending on where the researcher stands in the theory-generating process.

### Sample

Thirteen senior citizens living independently in the community and six visual instructors participated as data informants. The seniors were between 73 and 85 years of age, including seven women (75 to 85 years) and six men (73 to 84 years), and had participated in two previous studies of falling and visual impairment (Källstrand-Eriksson, Baigi, Buer & Hildingh, 2013; Källstrand-Eriksson, 2014). Two men and two women were living with their spouse, whereas nine were widows or widowers. Of the married couples, only one spouse in each couple was interviewed. Inclusion criteria were having a performance-based visual ability with mild vision loss or worse in best eye and/or visual field defects in the lower quadrants and having reported at least one fall according to data in the previous studies. Twenty-eight individuals met the inclusion criteria, and one participant at a time was randomly selected to be invited for an interview. First an invitation by letter was sent, followed by an invitation by telephone. All the seniors invited agreed to participate and were able to choose the venue for the interview.

The six visual instructors, five women and one man, came from the same county as the seniors and were invited by e-mail followed by a telephone call. Their job was to guide and support the visually impaired in the community, usually by visiting seniors in their homes. Since they meet a large number of visually impaired who also are at risk of falling because of the impairment, their knowledge about how seniors solve issues in daily life was considered valuable theoretical sampling. Their experience as visual instructors ranged between 6 months and 23 years.

### Data collection

We collected interview and observation data during December 2009 and January 2013. Twelve of the interviews and observations with the seniors took place in their own homes on one occasion. We wrote detailed field notes and collected audio recordings of what the participants said; we observed how they moved in familiar surroundings, noticing lighting and furnishing.

The formal interviews with the seniors lasted between 30 to 90 min and started with the question, “How do you perceive your daily life in relation to your vision and risk of falling?” Thereafter the participants talked openly. If something appeared to be of importance, clarifying questions were asked.

After the formal interviews the participants were often relaxed and continued talking about their daily life. All participants agreed to our use of this informal conversation as data in addition to the formal interview data. Directly after each interview further field notes of observations and informal talk were recorded in field notes. We interviewed one man over the telephone since he was too tired to manage a visit.

We interviewed two visual instructors at their offices and four by telephone for between 30 and 120 min. The visual instructors were initially asked, “What is your experience of working with visually impaired elderly people?” If needed, supplementary questions (mainly pertaining to the core variable) were asked, such as, “Can you elaborate on that?”, “Can you give me an example?”, or “Can you tell me about a case?”

### Generating the theory

We performed open coding immediately after the first occasion of data collection, comparing incidents to other incidents and later concepts to incidents and concepts to concepts (Glaser, 1978, 1998). This process finally led to the emergence of a tentative core category, after which we did selective coding of the data, analyzing incidents and concepts only related to the core. We wrote memos during the entire research process, starting with our first contact with the seniors by telephone. We eventually discovered the core category *self-preservation—*a concept that was enduring, had “grab” according to Glaser, and was connected to and explained most other concepts (Glaser, 1978, 1998). Thereafter, we interviewed the visual instructors as a further theoretical sampling of data associated with self-preservation or properties of self-preservation. After interviewing the six visual instructors, no new data emerged, and we reached theoretical saturation (Glaser, 1998). At this stage we analyzed and sorted memos into groups, comparing them with other groups of memos (Glaser, 1978). During this process of constant comparison, relationships between concepts arose and a theoretical model appeared. As a last step we tried matching different theoretical codes to the emerging model and wrote a first draft on a conceptual level avoiding details (Glaser, 2005). After writing the draft, we identified relevant literature with the purpose of positioning the emerging theory in an academic context (Glaser, 1998) and finally reached theoretical saturation (Glaser, 1978).

### Scientific rigor

There are four criteria for judging quality in CGT, which were taken into account regarding our study: fit, workability, relevance, and modifiability (Glaser, 1978, 1992, 1998). *Fit* means that the emergent categories represent the data they are meant to conceptualize (Glaser, 1978), and this goal is achieved through constant comparison instead of using preconceived codes and categories from an existing theory. *Workability* makes the core category recognizable and explains how the main concern is resolved (Glaser, 1998). In this study, the seniors’ behavior was interpreted and predicted by the core category. Another criterion is *relevance*, since the research has to be important, have “grab,” and not be only of academic interest (Glaser, 1978). The last criterion is *modifiability*. When the theory is modifiable, it may be used in other circumstances and new research, and it should be possible to fit new data entering the substantive area into the theory by modifying existing concepts. A grounded theory is neither right nor wrong; it just has more or less fit, relevance, workability, and modifiability. Readers of this grounded theory should evaluate it against these criteria.

### Ethical considerations

We applied the following ethical guidelines: the World Medical Association (2013) *Declaration of Helsinki—Ethical Principles for Research Involving Human Subjects* and the Swedish Act concerning the Ethical Review of Research Involving Humans (SFS 2003:460). We also obtained formal research ethics approval by the Regional Ethical Review Board in Lund, Sweden (566/2008). Information about the study was given both written and orally in advance. Written informed consent was obtained from each participant before enrollment. Participation in the study was voluntary and the participants could end their participation at any time without giving an explanation. All data were kept confidential and the participants were assured that identifying data was unavailable from unauthorized access, use, disclosure, modification, loss, or theft.

Consideration was taken with interviewing elderly individuals in their own homes when planning the interviews, but any risk of harm seemed negligible. Because the participants had already taken part in a previous study about their visual ability, the risk was considered small that they would feel forced to participate in another study. By giving thorough information about the study and making participation voluntary, harm was prevented.

The interviewer was supportive and took responsibility for making the participants feel comfortable talking about their daily lives in the interviewing situation. This measure was needed to avoid making the participants feel exposed and vulnerable, since they had both affected visual ability and at least one previous fall. After the interviews, contact information for the interviewer was supplied to the participants. When interviewing the visual instructors there was no risk for harm or discomfort, since the questions covered the visually impaired elderly persons they met in their profession. No information was asked that could identify the seniors as individuals, nor anything else making it possible to identify any of the other elderly individuals they met.

## Results

We discovered that seniors living independently with a visual impairment and at risk of falling want to remain the person they used to be by “self-preservation” with the dimensions “maintaining their established self” and “defying deterioration” (of their ageing self), the participants often uses both these approaches to preserve the self. The seniors maintain their established self by living in and with the past, keeping their home intact, retaining past activities and appearances by facading and avoiding burdening their family. They defy deterioration actively by moving (exercising, using walking aids, and driving); adapting by finding new ways; or networking by retaining old or finding new support networks of neighbors, friends or other groups of seniors. While maintaining one’s established self is mostly driven by inertia defying deterioration is a proactive and purposely driven strategy.

### Maintaining the established self

*Maintaining the established self* is about preserving the person one used to be or rather the life one used to have before age-related deterioration began. The seniors were living the life of the established self, found in memories of the past and in their homes. The social spaces were reduced, because many friends and relatives were dead and sometimes memories were all they had left.

#### Living the past

The seniors maintained the established self by *living the past*, dwelling on memories from times when they were stronger, worked, and enjoyed hobbies and family life. They kept up the established self by *reminiscing*: reliving and visualizing the past, enhancing the self, when characterizing the individuals they really were, hidden in aged bodies. With satisfaction, the seniors depicted journeys through adulthood and carefully told professional career stories, indicating a key part of their identities. An example of keeping up with the past is subscribing to a local newspaper from a region where they used to live. Articles, news, and information about friends and relatives created a sense of being present in the past.

*Living the past* was enhanced by *preserving the home* as it used to be, not changing much in the interior design. This point is critical because some seniors ignored suggestions for making fall preventive changes at home despite being aware of the risk reduction effect. Removing carpets, mats, and furniture was like removing memories and preserving the home made them feel secure. Widowers refused to make changes because they wanted to retain the memory of their deceased spouse. Each item or picture kept on significant shelves or in special drawers had its own story from the past, enhancing narratives and recalling memories. Many stories emerged of family gatherings, revealing close relationships with children, relatives, and friends.

Many rarely paid attention when stumbling in homely environments, claiming that they could move without any apparent hindrance, because they thought they knew every inch. Some were also reluctant to improve illumination as visual instructors recommended. Neither did they turn on the light when going to the bathroom at night because they thought they knew exactly where to go. They wanted to make their own choices and if a fall occurred, it was simply caused by their own negligence.

#### Facading

When becoming impaired, seniors *put up a facade* to hide the impairment instead of exposing their vulnerability, thus protecting their self-determination and their homes so nobody could take over its management. It was not always clear if they were aware of their fall risk, yet most were not worried about their visual impairment. Sometimes fall risk awareness was verbally expressed but, since daily life activities were kept constant, fall preventive measures involving changes were not taken. Even if they rationally knew they should take action, they did not act because of *change inertia*. One woman expressed that although she knew she ought to slow down her walking speed she could not do it and was unable to give a reason why. “I have always been rushing through life and still do … even though I know I do not have to. My husband always tells me to slow down and look where to put my feet when we are out walking” (woman, age 80). Because of change inertia, health risks in general and fall risk in particular remained high.

Even if some seniors needed support in daily life activities such as buying groceries, they often revealed an attitude of *burdening avoidance* towards family or friends. They wanted their children to use their spare time to be with their own families instead of supporting them. Although the participants did not want to ask friends or family openly, a wish for help was often subtly expressed.

The seniors also put up a facade when they did not want to look old and because of the misfit between body and mind—having a young mind in an aging body. As an example, they downplayed the risks of wearing improper shoes such as clogs and high heels in spite of relatives telling them that they might fall. They were aware of the risk when talking about it but wore the shoes anyway without giving it a thought. “My sons always tell me to wear proper shoes because of the risk of falling … but I have always worn clogs because I think they are comfortable and easy to put my feet in” (man, age 80).

The seniors accepted risks by being reluctant to use walking aids or white sticks displaying their visual impairment or old age when they were in different social contexts. A walking aid makes one look old and vulnerable. One man who exhibited great difficulty walking without a walking aid had a walker hidden in a corner of his bedroom. One woman fell badly and injured her back when not using a walking aide, but said she would act the same way again. By ignoring weakness, seniors were *juggling risk*, since it was often more important not to look old than to risk falling.

### Defying deterioration of the aging self

*Defying deterioration* means that seniors at risk of falling were engaged in behavior and taking actions that enhanced their capacities and preserved the self by compensating for bodily losses and deterioration that reduced their quality of life. By taking control of their lives, they felt independent and dignified in spite of a weakening body, since their minds often remain young. Moving, adapting, and networking are dimensions of defying deterioration of the aging self that clearly emerged in our data.

#### Moving

Moving involves *maintaining past activities*, such as driving despite apparent deteriorated function and holding on to hobbies, sports, and everyday work such as gardening and shoveling snow. These were ways of preserving the self that involved moving, but with increased health risks attached. Yet, the participants are not moving as much as they used to and also gardening is often done to a limited extent. Driving with impaired vision is evidently a risky behavior but for some seniors driving is important to defy deterioration since the increased mobility takes them to places and situations where their self is better preserved. Moving is also done by *using mobility aids* such as walkers, sticks, and electric scooters facilitates daily life activities such as doing errands and buying groceries and is also a way to overcome a tendency to stick to everything old and familiar. A walker may also be used to sit on while having a chat or taking a break resting one's legs. Being able to go wherever and whenever you want without being reliant on someone else is a self-relying freedom. The use of mobility aids often requires an incremental learning process, eventually charged with stigma that has to be overcome. A trick to convince one's established self to use mobility aids or other tools is to render the objects human properties or to decorate them in personal ways. Seniors thus anthropomorphized the mobility aids, calling their walker “my fiancée” or “my boyfriend.” This strategy may reduce stigma and the odd feelings that accompany recognition of aging and deteriorated function.

When performing various moving activities such as dancing, not only the socialization is important for defying deterioration, but also the physical activity since it keeps the body fit. Another way of staying fit is by walking, either with or without walking aids. In slippery walking conditions, the seniors were more careful than they used to be when they were younger. For some seniors, walking the dog was their only physical activity. “If it wasn't for my dog I'd be sitting on my sofa doing nothing” (man, age 81).

#### Adapting

Adapting activities to visual deterioration can include new ways of doing familiar things, such as listening to audiobooks and audio newspapers instead of reading, or finding new ways of cooking without being able to see the handles of the pots and pans. One woman who loved to watch all kinds of sports on TV adapted to listening to sports on the radio because of her visual deterioration. This adaptive “prompting” of sport events was actually illustrative and exciting, since she got a more detailed description. Another example of adaptive prompting occurs when a partner whispers to the senior with visual impairment who they meet when out walking.

#### Networking

The seniors retained their younger identities as a way of defying deterioration by nurturing mutual support networks of friends, family, and other acquaintances. This involved looking after grandchildren or great-grandchildren and taking care of pets when pet owners were working. Being needed was a way of making life meaningful, and working after retirement was a way of defying deterioration by sustaining a networking activity that comes naturally to some seniors even past the age of 80. Self-employed seniors enjoyed working to a limited extent, even if their businesses had been taken over by their children.

Networking by developing new support networks is dependent on the physical condition and ability of the seniors to contribute, yet all the participants in this study took part in various new social activities. Being part of new social contexts is a way of networking that defies deterioration and upholds a sense of belonging. It does not matter whether the activities are organized or spontaneous. Social activities can be a small chat with another person at a bench, voluntary work, or a senior dance at the community center. “You do not have to be alone even though you are old and it is never too late to meet a new partner” (widower, age 83, participating in senior dances).

## Discussion

The senior participants in our study had some kind of visual impairment and had fallen at least once. Yet the risk of falling did not worry them much. Instead, their main concern was to stay the same person as they used to be, despite getting older. This core pattern is called *self-preservation* and has two dimensions: *maintaining the established self* and *defying deterioration*. Maintaining the established self was a self-preservation strategy typically driven by change inertia. It included living in the past, keeping an intact home, and retaining past activities and appearances by facading and avoiding burdening family members. Defying deterioration involved more action from the participants who were trying to be mobile (exercising, using walking aids), adapt (finding new ways to perform familiar activities), or network (sustaining old support networks and finding new ones).

The seniors we met were risk-indifferent, meaning that they were not very concerned about risks such as falling. They expressed awareness of their aging bodies and the accompanying risks, but did not take much action or change their conduct to reduce risks. On the contrary, strategies for maintaining the established self were often associated with a risk increase.

Other research aligns with the conclusions from our theory of self-preservation, with seniors maintaining their independence and autonomy, perceiving themselves as capable individuals who make their own decisions (Yardley et al., 2006), and therefore possibly denying that they actually are old and vulnerable. They consider falls as a consequence of aging and therefore both natural and unavoidable (Roe et al., 2008; Yardley et al., 2006). Some seniors accept that the risk of falling is present all the time and consider fear of falling a waste since falls often are unpredictable. Many seniors who live independently perceive their fall risk as low (Hill et al., 2011) even though they have a relatively high risk of falling caused by age-related physiological factors (Delbaere, Close, Brodaty, Sachdev, & Lord, 2010). They have a relatively high quality of life and a positive outlook on life in spite of their risk. Many seniors maintain their independence by taking actions that lead to autonomy and well-being, regardless of the fact that the risk of falling is not reduced (Yardley et al., 2006). This may be the reason why the seniors in our study were not concerned about the risk of falling, even though they all had fallen at least once.

Defying deterioration is a proactive strategy to preserve the self and involves initiatives to adapt and make new ways of dealing with daily life activities, as confirmed by other research (Reichstadt, Sengupta, Depp, Palinkas, & Jeste, 2010). The seniors in our study promoted their independence and autonomy, perceiving their minds as young despite aged bodies. They were trying to maintain an equilibrium between activity and well-being that sometimes turned into risky behavior. Another study showed that continuing activities and social relations, even though at a new level because of age, imparts strength and confidence (Hörder, Frändin, & Larsson, 2013). This resembles the healthy aging movement views that highlight the relationship between activities, place, well-being, and healing (Cutchin, 2005).

The seniors in our study wanted to remain who they were by maintaining the past as a way to connect with their earlier lives. The “elderly self” was interpreted as being related to a continuum of earlier life in a grounded theory study of 12 white United Kingdom citizens aged 70 to 92 (Tanner, 2001); and in a South Korean in-depth interview study of 34 participants joy in the present was related to the past (Shin, Kim, & Kim, 2003).

*Protecting the core self* was of great importance to seniors in a previously cited study (Tanner, 2001)—a pattern analogous to *maintaining the established self* in our study. Reminiscing is a way of maintaining the self that may also be expressed as a review of one's life, which may lead to inner strength, accomplishment, and satisfaction with “one's life today,” as seen in a study on “successful aging” from California (Reichstadt et al., 2010).

One reason why the seniors in our study were not concerned about their fall risk or impaired vision might be that they thought fall prevention was a concern for elderly and disabled people and not for themselves (Yardley et al., 2006). The seniors refused to accept their high age, exposing themselves as old and vulnerable, and by not using aids they were keeping a younger and healthier facade. Thereby they were resisting threats to their identity (Tanner, 2001), since they thought their body was younger than their chronological age (Kleinspehn-Ammerlahn, Kotter- Gruhn, & Smith, 2008; Westerhof, Krauss Whitbourne, & Freeman, 2012) and therefore wanted their physical appearance to match their young mind as a way of preserving the self.

The phenomenon of people considering themselves young even if their body is old has been seen as an obstacle for overcoming risky behavior in other studies as well (Berlin Hallberg, Albertsson, Bengtsson, Dahlberg, & Grahn, 2009; King & Farmer, 2009). When a mind is trapped in an aged body, there is no balance between body and mind and conflicts appear (Reichstadt et al., 2010), sometimes caused by visual impairment. In spite of the imbalance between mind and body, some seniors mostly maintain their past activities, while others are better at using aids or adapting by changing habits and/or behaviors. Yet adaptation is not always a matter of the senior's choice (Tanner, 2001). The aging body might force them to adapt, even though the adaptation is a threat to the self. In such situations, support from both family and/or healthcare providers might be a way for the seniors to accept the need to adapt and to discover new means of self-preservation, instead of getting stuck in old patterns. It therefore seems important to let seniors find their own ways to a good life instead of telling them what to do or not to do.

A feeling of safety from being at home and in familiar environments is indeed a paradox, since self-preservation by maintaining the established self means that the seniors were exposing themselves to risks at home. Emotional hurdles to change made it difficult to adjust their homes by removing obstacles such as rugs to prevent falls. Many of the seniors wanted to maintain a certain domestic standard since their homes reflected them as individuals, representing continuity from their earlier life (Tanner, 2001). They had strong attachments to their homes, mentioning how they loved it, how the sun lit it up, describing their garden or their favorite chair (Wiles et al., 2009). Through the memories of places, experiences, and feelings there was a link between the past and present that has been described as a way of creating and maintaining a sense of wholeness in life (Manzo, 2005). The home and neighborhood were also a connection with the past that most people were emotionally attached to because of family and friends (Tanner, 2001; Wiles, 2005). This connection gave a potentially misleading sense of security and warmth (Roe et al., 2008; Yardley et al., 2006).

Even though the seniors in our study knew that their bodies were aging, “behavioral inertia” made them continue with the ways of life as they used to. One could argue that this is normal and nothing to be surprised about. It is not abnormal to do what you have always done. However, from a health perspective seniors have higher risks of all kinds of health issues, including trauma-related disorders. In addition, knowing that these risks are highly dependent on lifestyle factors makes the normal reminiscing actions ruled by inertia appear as medically risky behavior. Behavioral inertia was confirmed in a South Korean study where seniors expressed reluctance to change (Shin et al., 2003).

When aging friends and family members move to nursing homes or die, the social spaces get smaller, but being socially active in old or new networks is considered valuable (Hörder et al., 2013; Tanner, 2001; Wiles, 2005). There are, however, seniors who choose to be alone. Some seniors in our study were satisfied with their shrinking social networks and thought the memories of the past were sufficient. Others did not want to be a burden to their children, who were busy with their own lives or lived far away. This self-limiting could pose a threat to identity and reduce self-preservation by depriving them of the pride of having a family and being part of a social context with friends, a home, and a neighborhood (Hörder et al., 2013; Tanner, 2001; Wiles, 2005).

Although visual impairment made driving difficult, some seniors in our study drove anyway, thereby jeopardizing their own and others’ safety by risk denial or risk juggling. If they stopped driving, their social space would shrink and they would risk losing independence (Wiles et al., 2009). Today there are ways of social networking other than physical, since the world is shrinking with the Internet as a ubiquitous resource.

Using aids not only could help to break social isolation and prevent falls, but also could promote independence and control (Berlin Hallberg et al., 2009; McInnes & Askie, 2004). Therefore, a major issue is to communicate fall prevention in a way that concerns the seniors. Paternalistic approaches where experts and authorities plan and perform interventions without establishing them among seniors are common today (Swedish Institute of Public Health, 2007), causing barriers because the actions are seen as insulting (Yardley et al., 2006). Involving seniors in decision-making and planning to figure out relevant actions is therefore crucial (Berlin Hallberg et al., 2009).

The data collection and analysis method used in this study —CGT—made the seniors’ main concern emerge from the data, explaining their behavior in a conceptual way (Glaser, 1998). Using both observations and interviews is a strength with this study, especially since CGT encourages the use of different types of data. After the formal interviews, the seniors were often relaxed and informal conversations took place in their hallways, yielding useful data. This shows the importance of field notes and informal data, since valuable data would have been lost if our data collection had relied on recorded data only.

Even though some claim that in CGT no preconceptions are allowed, the researcher always has his or her own perspectives. Therefore, Glaser recommends that the researcher interview herself or himself; once aware of his or her own perspectives the researcher may use them as data to be coded and compared just like the rest of the data when generating a conceptual and abstract theory that explains the participants’ behavior (Glaser, 1998). In this study the researchers’ preconceptions of fall risk were not used as data since the participants did not consider their risk of falling and visual impairment as a concern and a bias due to preconceptions therefore did not occur.

In summary, the knowledge generated in this study gives health care providers a rich understanding of self-preservation as senior citizens’ way of dealing with their main concern of *being who they used to be*. Their daily life was influenced by visual impairment and a risk of falling, but this was not a major concern for the seniors. Our theory can hopefully help health care providers in general better understand the complexity of the situation when promoting health preventive actions that could be informed by essential knowledge of seniors’ coping activities provided in this paper. Moreover, self-preservation is generic human behavior, and modifying this theory to other fields could therefore be of interest for future research.
